# Development and validation of whole-genome SSR markers in sugar beet (*Beta vulgaris* L.)

**DOI:** 10.3389/fpls.2026.1873607

**Published:** 2026-08-11

**Authors:** Chengcheng Yang, Shengnan Li, Zhi Pi, Zedong Wu, Yongli Hao

**Affiliations:** 1 College of Modern Agriculture and Ecological Environment, Heilongjiang University, Harbin, Heilongjiang, China; 2 Chifeng Academy of Agricultural and Animal Husbandry Sciences, Chifeng, China

**Keywords:** genome-wide, polymorphic primers, resequencing, simple sequence repeat markers, sugar beet

## Abstract

Sugar beet (*Beta vulgaris* L.) is an important sugar and cash crop worldwide. To systematically characterize SSR (Simple Sequence Repeat) loci across sugar beet chromosomes and enable the precise identification of germplasm resources, this study conducted a genome-wide scan for SSR loci, analyzed their distribution patterns, and determined their genotypes using resequencing data from 123 sugar beet varieties. The results revealed an abundance of SSR loci in the sugar beet genome, with a total of 135, 379 identified, from which 135, 344 pairs of SSR primers were designed (135, 344 primer pairs successfully designed; 35 loci failed to meet design criteria). Specifically, 31, 748 primer pairs were designed based on SSRs located in unassigned scaffolds, and 103, 596 primer pairs from SSRs assigned to the nine chromosomes. Through bioinformatic analysis, we identified 28, 768 SSR primers located in multi-copy genes with PIC (Polymorphism Information Content) ≥ 0.5, and 2, 326 SSR markers located in single-copy genes residing in various genic regions (among which 543 had PIC ≥ 0.5, with the highest reaching 0.776). PCR (Polymerase Chain Reaction) validation confirmed 20 robust and polymorphic markers producing clear and reproducible bands. Among them, 10 SSR primers located in multi-copy genes exhibited three or more polymorphic types, and 10 markers located in single-copy genes displayed 2–3 polymorphic types. The most polymorphic marker, YCD-4-2, detected 11 polymorphic types across 48 varieties. Furthermore, to explore markers with potential functional significance, we annotated the genes harboring SSR markers located in single-copy genes. The results showed that 1, 264 SSRs located in single-copy genes were localized to 967 genes, which are significantly enriched in pathways related to carbohydrate metabolism, stress responses, and plant-pathogen interactions. The 20 validated markers and the 2, 326 SSRs located in single-copy genes provided in this study can be directly applied to fingerprinting of sugar beet varieties, seed purity testing, and marker-assisted selection, thus representing a practical resource for molecular breeding.

## 1 Introduction

New evidence indicates that the progenitor of beet originated from Beta vulgaris ssp. maritima (sea beet) in Greece, a plant species native to the coasts from Morocco to the North Sea and the Mediterranean, with beet cultivation in the Mediterranean region dating back 10, 000 years (Felkel et al., 2023; Sandell et al., 2022). Furthermore, sugar beet shares a common palaeotriploid ancestor with Rosaceae and Asteraceae (Wang L. et al., 2023).

During the last 200 years of sugar beet breeding, sugar content has been improved from ~8% to ~18% in modern cultivars through sustained breeding efforts, which have also targeted disease resistance, taproot yield, monogermy, and bolting resistance (Felkel et al., 2023). It is widely accepted that beet was initially consumed as a leafy vegetable. The domestication of the swollen storage root likely began in the Near East (Iraq, Iran, Turkey). Subsequently, this trait and the associated beet types spread westward along trade routes, eventually reaching Egypt (earliest archaeological evidence, ~3500 BCE) and further into Europe (Goldman and Janick, 2021). Today, in addition to sugar production, the whole beet plant and its by-products (including green leaves, molasses and pulp residues) can be used as animal feed or as raw material for alcohol production, and can even be converted into bioethanol (Puligundla and Mok, 2021; Yolcu et al., 2022). Sugar beet (*Beta vulgaris* L.) ranks as one of the world’s most important industrial crops, providing approximately 21% of global sugar production, second only to sugarcane (USDA, 2023). Moreover, sugar beet exhibits cold tolerance and alkali resistance, which contribute to its economic value. The first sugar beet processing plant in China was established in Heilongjiang Province in 1905, and sugar beet was introduced into the country on a large scale in 1906 (Li et al., 2025). Currently, the dominant sugar beet production regions in China are located north of 40°N, including Northeast, North and Northwest China, among which North China has the largest cultivation area, accounting for approximately 65% of the total sugar beet acreage in China.

The completion of the sugar beet genome sequence in 2014 provided a key foundation for whole-genome molecular marker development, functional gene mapping and comparative genomics studies (Mandal et al., 2018). With the advancement of bioinformatics, molecular marker technologies have been widely applied in plant genetic map construction, genetic diversity analysis, purity and marker-assisted selection (Panahi et al., 2024). SSR (simple sequence repeat), also known as microsatellite DNA, is a PCR-based molecular marker technology using specific primers. SSRs are tandem repeat sequences composed of short nucleotide motifs (typically 1–6 bp) repeated in succession to span loci of up to several dozen base pairs. Owing to their low equipment requirements and high operability, SSRs have remained a popular direction in the field of molecular marker development (Shavrukov et al., 2024). However, SSR markers are species-specific, making the development of marker loci a major challenge for their application, and the lack of SSR markers has severely limited the progress of related research. Given the distinct sequence characteristics of SSR loci and the increasing availability of various types of sequence information, bioinformatics-based mining of SSR loci has emerged as a novel approach for marker development (Geethanjali et al., 2024).

Because China is not a centre of origin for sugar beet, it lacks wild germplasm resources and exhibits low genetic diversity. Consequently, the collection and utilization of sugar beet germplasm resources are particularly important. However, the large number of accessions complicates research and utilization by breeders. To deepen the understanding of the composition of genetic diversity and the characteristics of collected resources, improve the utilization of germplasm, and broaden the genetic background of new sugar beet varieties, the establishment of a core collection is of great significance (Dohm et al., 2014; Li et al., 2025).

To efficiently develop SSR markers, this study employed 123 foreign sugar beet varieties previously resequenced in our laboratory to systematically carry out genome-wide SSR marker discovery and validation. The objectives of this study were: to comprehensively identify SSR loci across the sugar beet genome and characterize their distribution; to screen for highly polymorphic SSR primers located in multi-copy genes suited for rapid germplasm identification; and to prioritize SSR markers located in single-copy genes, thereby providing locus-specific markers for QTL mapping and marker-assisted selection. In addition, we performed functional annotation and pathway enrichment analysis to investigate the biological context of these SSRs located in single-copy genes.

## 2 Materials and methods

### 2.1 Plant materials

A total of 123 foreign-introduced sugar beet varieties from six breeding companies were used in this study. These 123 varieties encompass all varieties cultivated in China and do not include wild or semi-wild species. They were provided by the Key Laboratory of Sugar Beet Molecular Genetics and Breeding, Heilongjiang University, and were planted in May 2024 at the Experimental Base of Heilongjiang University in Hulan District, Harbin (45°59′50.291″N, 126°38′25.292″E). The varieties comprised 31 accessions from Germany (14 from KWS SAAT SE and 17 from Strube), 17 from Lion Seeds Ltd. (UK), 43 from SES VanderHave (France), 25 from MariboHilleshög Aps (Denmark), and 7 from BETASEED (USA).The information of 8 tested sugar beet varieties used for SSR primer screening is shown in **Table 1**. The numbers, names and breeding companies of the sugar beet varieties used in this study are listed in **Supplementary Table 2**.

**Table 1:** Eight varieties of sugar beets that were used for SSR primer screening.

Number	Variety Name	Breeding Company	Number	Variety Name	Breeding Company
1	MA3	MariboHilleshög ApS	5	MA18	MariboHilleshög ApS
2	KWS7772	KWS SAAT SE	6	L2306	Lion Seeds Ltd.
3	SX1537	SES VanderHave	7	BTS6990	BETASEED
4	MK4250	SES VanderHave	8	ST13915_1	Strube

### 2.2 Experimental methods

#### 2.2.1 Genome resequencing and SSR locus identification

Young leaves from 123 sugar beet samples were collected. Genomic DNA was extracted using the cetyltrimethylammonium bromide (CTAB) method. DNA concentration and purity were measured using a NanoDrop 2000/2000c spectrophotometer (Thermo Fisher Scientific), and samples were placed on dry ice and sent to Majorbio Bio-Pharm Technology Co., Ltd. for Illumina next-generation sequencing.

#### 2.2.2 Whole-genome resequencing experimental procedure

After genomic DNA passed quality inspection, it was randomly fragmented by ultrasonication. The fragmented DNA was subjected to end repair, 3’ adenylation, and adapter ligation. Target fragments of approximately 350 bp were enriched by magnetic bead adsorption, followed by PCR amplification to construct the sequencing library. Following library quality control, the qualified libraries were sequenced on the Illumina NovaSeq X Plus™ platform using the paired-end 150 bp (PE150) strategy, generating a total read length of 300 bp.

After sequencing, raw image data were converted into sequence data (stored in FASTQ format) through base calling. Raw reads containing adapter contamination, low overall quality, and poor-quality bases at the ends were filtered to obtain high-quality clean data. The clean reads were then aligned to the sugar beet reference genome RefBeet-1.2.2 using the BWA-MEME algorithm (Jung and Han, 2022), producing BAM files.We used 10x coverage to analyze the allele frequencies of various SSRs within the population. This is an economical and efficient approach that is suitable for SSR typing at the population level, consistent with (Willems et al., 2017).

#### 2.2.3 Genome-wide mining of SSR loci

Based on the sugar beet reference genome (RefBeet-1.2.2), we used the Misa (MIcroSAtellite identification tool) v2.1 software (Wang H. et al., 2023) run with Perl to identify and classify genome-wide SSR loci. The parameters in the misa.ini configuration file were set as follows: mononucleotide repeats with a minimum of 7 repeats; dinucleotide repeats with a minimum of 4 repeats; trinucleotide, tetranucleotide, pentanucleotide and hexanucleotide repeats with a minimum of 3 repeats; and the minimum interval between two SSRs was set to 100 bp, with two SSRs considered as a compound SSR if the interval exceeded 100 bp. Finally, the physical coordinates, repeat motifs and repeat numbers of all genome-wide SSR loci were obtained.

#### 2.2.4 SSR primer design and polymorphism analysis

Based on the SSR locus information identified by MISA, we designed SSR primers in batches using Primer3. Forward and reverse amplification primers were designed following standard primer design criteria, which included: GC content of primers between 20% and 80%; primer length of 18–27 bp, with 20 bp being optimal; annealing temperature range of 55 – 70 °C; and amplification product length between 50 and 150 bp. During design, we avoided having an A at the 3′ end and prevented the formation of potential secondary structures.The 50–150 bp amplicon size range was adopted for several technical reasons. First, the 150 bp paired-end sequencing strategy employed in this study is inherently suited for detecting SSR loci within this size window, as individual reads can fully span the repeat region and its flanking sequences. Second, short amplicons reduce the likelihood of harboring additional SSR motifs or sequence variants within the amplified fragment, thereby minimizing genotyping complexity and improving locus specificity. Third, shorter amplification products generally exhibit higher PCR efficiency and primer-binding specificity, which facilitates high-throughput genotyping and enhances cross-laboratory reproducibility. It should be noted that the polymorphism of an SSR marker is determined by variation in the copy number of its core repeat unit rather than by the total amplicon length; consequently, short-amplicon markers can be equally or even more informative than their longer counterparts.

**Figure 1:**
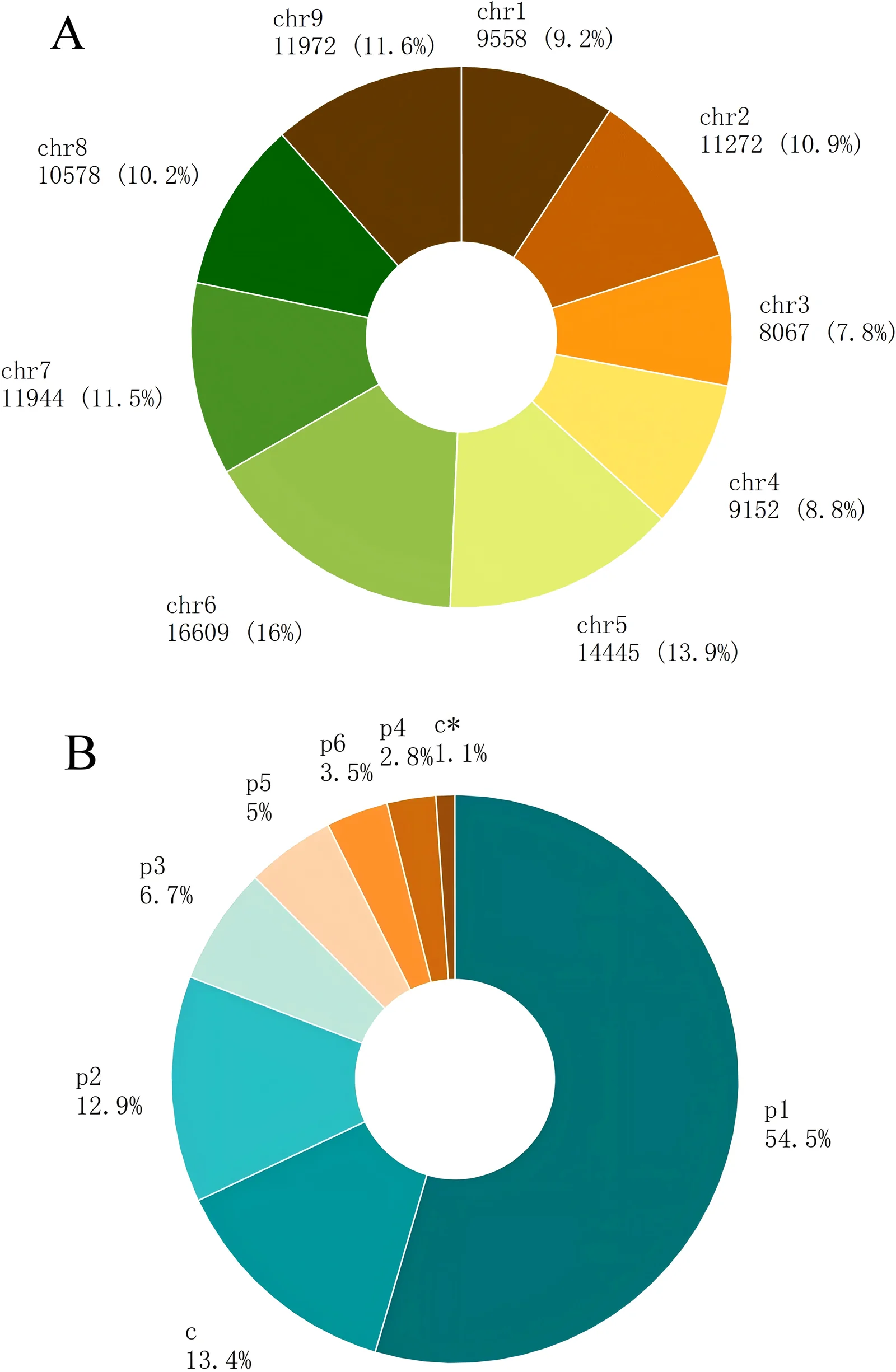
Distribution of SSR loci and base type statistics in the sugar beet genome; **(A)** Distribution of SSR Loci Across Nine Chromosomes; chr1 - 9: Represent chromosomes 1 to 9, respectively. **(B)** Quantity and Proportion of SSRs Classified by Repeat Type. p1 - p6: represent repeats of 1 to 6 bases, respectively; c: compound SSR marker; c*: imperfect SSR marker.

Based on the genotyping results from HipSTR, we organized the genotype data of each sample using a Python script and converted them into CSV (Comma-Separated Values) format compatible with Cervus 3.0.7. The observed heterozygosity (Ho), expected heterozygosity (He) and polymorphism information content (PIC) for each SSR locus were calculated using Cervus 3.0.7 software. “Origin” 2024 was used to generate **Figures 1A, B**.

#### 2.2.5 Identification of intra-species single-copy and multi-copy genes

For marker-assisted selection, markers located within low-copy or single-copy genes are preferred as they provide unambiguous chromosomal addresses. Therefore, in this study we performed a whole-genome similarity search using Diamond software (Buchfink et al., 2026) to perform an all-against-all alignment of all protein sequences from the sugar beet reference genome (RefBeet-1.2.2) against themselves (Meng et al., 2021). The parameters were set as follows: E-value threshold ≤ 1e-5, sequence identity ≥ 40%, and the output format was set to format 6 (BLAST tabular format).The threshold of 40% sequence identity for defining single-copy genes was adopted following Addou et al (Addou et al., 2009), who demonstrated that above 40% identity, functional conservation among homologous domains reaches >90% confidence. In sugar beet, a paleotriploid species, this threshold effectively distinguishes recent duplicates (which retain high identity) from ancient paralogs (which have diverged further). Habermann (2016) noted that two proteins are considered remote orthologs if they originated from a speciation event and share less than 30% sequence identity at the protein level.Below this threshold lies the twilight zone of sequence identity, a term first used by Doolittle in 1986 (Doolittle 1986) to describe a threshold in sequence similarity, at which two proteins are hard to align. Therefore, 40% was selected as the lowest safe threshold above the twilight zone, balancing sensitivity for detecting true single-copy genes against the risk of including paleotriploid-derived paralogs. Higher thresholds (e.g., 50% or 60%) would excessively filter out genuine single-copy orthologs, reducing the number of usable SSR markers. We wrote a Python script based on the Pandas library to integrate the list of single-copy genes with the genome-wide SSR loci information. The script performed the following operations: (1) parsed locus identifiers (format: chromosome-position) from the single-copy gene annotation file; (2) loaded the chromosome coordinate file containing the identified SSR loci; (3) performed an intersection operation between the candidate regulatory regions (from 2 kb (Xue et al., 2024) upstream of the transcription start site to 2 kb downstream of the transcription termination site, an interval typically containing core promoters and key cis-acting regulatory elements) and the physical coordinates of the genome-wide SSR loci. As a result, a total of 2, 326 SSR loci residing within single-copy gene regions and their flanking regulatory regions were identified, with the remainder constituting SSR primers located in multi-copy genes.

It is important to clarify the scope of this classification. The pipeline described above identifies SSR loci that are physically located within or adjacent to genes classified as single-copy based on protein-level similarity searches using Diamond. This classification reflects gene copy number at the protein level rather than marker copy number at the whole-genome DNA level. An SSR located in a single-copy gene may still possess homologous flanking sequences elsewhere in the genome—in non-coding regions, pseudogenes, or unannotated loci—that would not be detected by protein-based similarity searches. Therefore, the term “SSR markers located in single-copy genes” is used throughout this study to accurately describe the classification basis. Experimental verification of single-locus amplification, such as genome-wide BLAST searches of flanking sequences or segregation analysis in biparental populations, would be required to confirm that a given marker amplifies a single genomic locus.

#### 2.2.6 Functional annotation and pathway enrichment analysis

To investigate the biological processes and pathways associated with this gene set, we utilized the data from a GTF file—including gene ID, transcript ID, chromosome location, start position, and end position—to obtain the annotation results for all transcripts against various databases. A species-specific OrgDb database was constructed using the AnnotationForge package in R. Subsequently, Gene Ontology (GO) enrichment analysis and Kyoto Encyclopedia of Genes and Genomes (KEGG) pathway analysis were performed using the clusterProfiler package (version 4.6.2).

#### 2.2.7 SSR primer selection criteria

To select candidate primers for experimental validation, 48 SSR primer pairs were chosen based on the following criteria:

1. Even distribution across the nine chromosomes (approximately 17–18 primers per chromosome);
2. PIC ≥ 0.5 as predicted from the resequencing data;
3. Inclusion of both SSR primers located in multi-copy genes and SSRs located in single-copy genes;
4. Coverage of di-, tri-, tetra-, penta- and hexanucleotide repeat motifs;
5. Amplicon size differences of at least 10 bp to facilitate gel resolution;
6. Preferential selection of SSR loci with six or more predicted alleles based on the resequencing data from the most diverse accessions.
7. Only SSR markers detected in more than 50% of individuals were retained for subsequent analysis.

#### 2.2.8 PCR validation of SSR marker polymorphism

The amplification reaction mixture had a total volume of 5 μL and contained: 0.2 μL each of forward and reverse primers, 1 μL of DNA template (20 ng/μL), 1.1 μL of ddH_2_O, and 2.5 μL of 2× Rapid Taq Master Mix (Beijing Juhemei Biotechnology Co., Ltd., Beijing, China). PCR was performed using a Thermo Fisher 96-well thermal cycler (Thermo Fisher Scientific™, Shanghai, China).

The PCR amplification program was as follows: initial denaturation at 94 °C for 3 min; followed by a touchdown PCR program consisting of 15 s denaturation at 95 °C, 15 s annealing at 65 °C for 2 cycles per temperature step, with the annealing temperature decreasing stepwise to 56 °C, and 30 s extension at 72 °C; then 25 cycles of 15 s denaturation at 94 °C, 15 s annealing at 55 °C and 30 s extension at 72 °C; and a final extension at 72 °C for 5 min. The amplified products were stored at 4 °C.

PCR products were detected by 8% non-denaturing polyacrylamide gel electrophoresis. After electrophoresis, the gels were stained with G-red fluorescent nucleic acid dye (BioTeke Corporation, Beijing, China), visualized using a gel imaging system, and photographed for subsequent data analysis. Primers that produced no bands, non-specific bands, or inconsistent amplification were discarded. Finally, SSR markers that amplified stably and exhibited two or more polymorphic patterns were selected. “polymorphic patterns” refers to the distinct banding patterns observed for a given SSR marker across different varieties, representing different allele sizes at that locus.

#### 2.2.9 Data statistics and analysis

The results of molecular marker detection are determined by the 0/1 assignment method. Initially, the amplified bands were manually analyzed carefully and the corresponding results were recorded. Subsequently, in order to generate a binary data matrix consisting of 0s and 1s, when a band appeared at the same position, it was assigned a value of “1”, and when there was no band, it was assigned a value of “0”. The genetic diversity indicators were calculated using Popgene 1.32, including Ho (observed heterozygosity), He (expected heterozygosity), Ae (effective number of alleles), Ar (allele richness), and I (Shannon’s information index). PIC (polymorphism information content) was calculated using PowerMarker 3.25.

## 3 Results and analysis

### 3.1 Sequencing data alignment results

Using the Illumina NovaSeq X Plus™ platform, 123 foreign sugar beet varieties were resequenced (**Table 2**). A total of 252 GB of raw sequencing data were obtained from this sequencing run, with an average of 21, 321, 454 reads per sample, corresponding to a total of 6, 439, 079, 101 bases.After quality control filtering to remove adapters and low-quality sequences, the average sequencing depth of the samples reached 13.01× relative to the reference genome, and the average genome coverage obtained after aligning the filtered clean reads of all samples was 87.36%.

**Table 2:** The statistics of the average values of sequencing data for sugar beet varieties fromsix companies.

Breeding Company	Original sequencing reads	Original sequencing base	After filtering Q30 (%)	After filtering reads	The filtered bases	Sequencing depth	Coverage rate (%)
Betaseed	21875023	6606257075	96.1	21724983	6509661140	13.27	86.99
KWS	21497840	6492347745	96.1	21355757	6399081349	13. 12	87.18
Lion	20669994	6242338188	96.2	20530992	6154280856	12.6	87. 14
Maribo	21149728	6387217723	96.23	21006148	6296611096	12.91	87.17
SES	21486036	6488782928	96.3	21342632	6401934697	13. 12	87.6
Strube	21435957	6473659014	96.18	21293795	6388094147	13.07	87.58
Total	21321454	6439079101	96.22	21178567	6350191962	13.01	87.36

### 3.2 Characteristics of SSR loci in the sugar beet genome

A total of 135, 379 SSR loci were identified in the sugar beet genome. Among them, chr6 (16, 609 loci) contained the highest number of SSR loci, whereas chr3 (8, 067 loci) contained the lowest (**Figure 1A**).The remaining 31, 748 SSR loci were located on unassigned scaffolds and were excluded from chromosomal distribution analysis.

Among all SSR loci, mononucleotide repeats were the most abundant, with 73, 832 loci accounting for 54.54% of the total. Dinucleotide and trinucleotide repeats were the next most abundant, with 17, 417 and 9, 070 loci, representing 12.87% and 6.70% of the total, respectively. Tetranucleotide, pentanucleotide and hexanucleotide repeats together accounted for 11.36% of all SSR loci, with 3, 763, 6, 828 and 4, 795 loci, corresponding to 2.78%, 5.04% and 3.54% of the total, respectively. In addition, compound SSRs (two SSRs separated by<100 bp) and imperfect SSRs together comprised 19, 674 loci, accounting for 14.53% of the total (**Figure 1B**).

### 3.3 Genome-wide development of SSR primers in sugar beet

Based on the 135, 379 SSR loci identified in the sugar beet reference genome (RefBeet-1.2.2), a total of 135, 344 primer pairs were successfully designed using Primer3, representing a success rate of 99.97%. The remaining 35 loci failed to meet the primer design criteria because their flanking sequences were too short or they were located at scaffold ends. Among these, 103, 596 primer pairs were located on the nine chromosomes of sugar beet; primer pairs that were not assigned to chromosomes were excluded from statistical analysis. The overall frequency of designed primers was 275.1 pairs per Mb, with chr6 having the highest number of SSR primers (16, 609) and chr3 having the lowest (8, 067).

Among individual chromosomes, chr3 showed the highest SSR primer frequency, with approximately 303.6 primer pairs per Mb and an average interval of 3.29 kb between adjacent SSRs; chr9 showed the lowest frequency, with 264.4 primer pairs per Mb and an average interval of 3.78 kb (**Table 3**).

**Table 3:** Statistics of the Length, Number of Primers, Frequency (per Mb), and Average Interval (kb) of the 9 Chromosomes of Beet.

Chromosome	Length (bp)	Number of SSR primers	Frequency (per Mb)	Average distance (kb)
1	34,941,034	9,558	273.5	3.66
2	40,393,389	11,272	279.1	3.58
3	26,577,699	8,067	303.6	3.29
4	33,024,243	9,152	277.2	3.61
5	52,461,754	14,445	275.4	3.63
6	60,962,716	16,609	272.4	3.67
7	44,152,522	11,944	270.6	3.70
8	38,796,167	10,578	272.7	3.67
9	45,274,173	11,971	264.4	3.78

### 3.4 Chromosomal distribution analysis of SSR primers located in multi-copy genes

**Figure 2:**
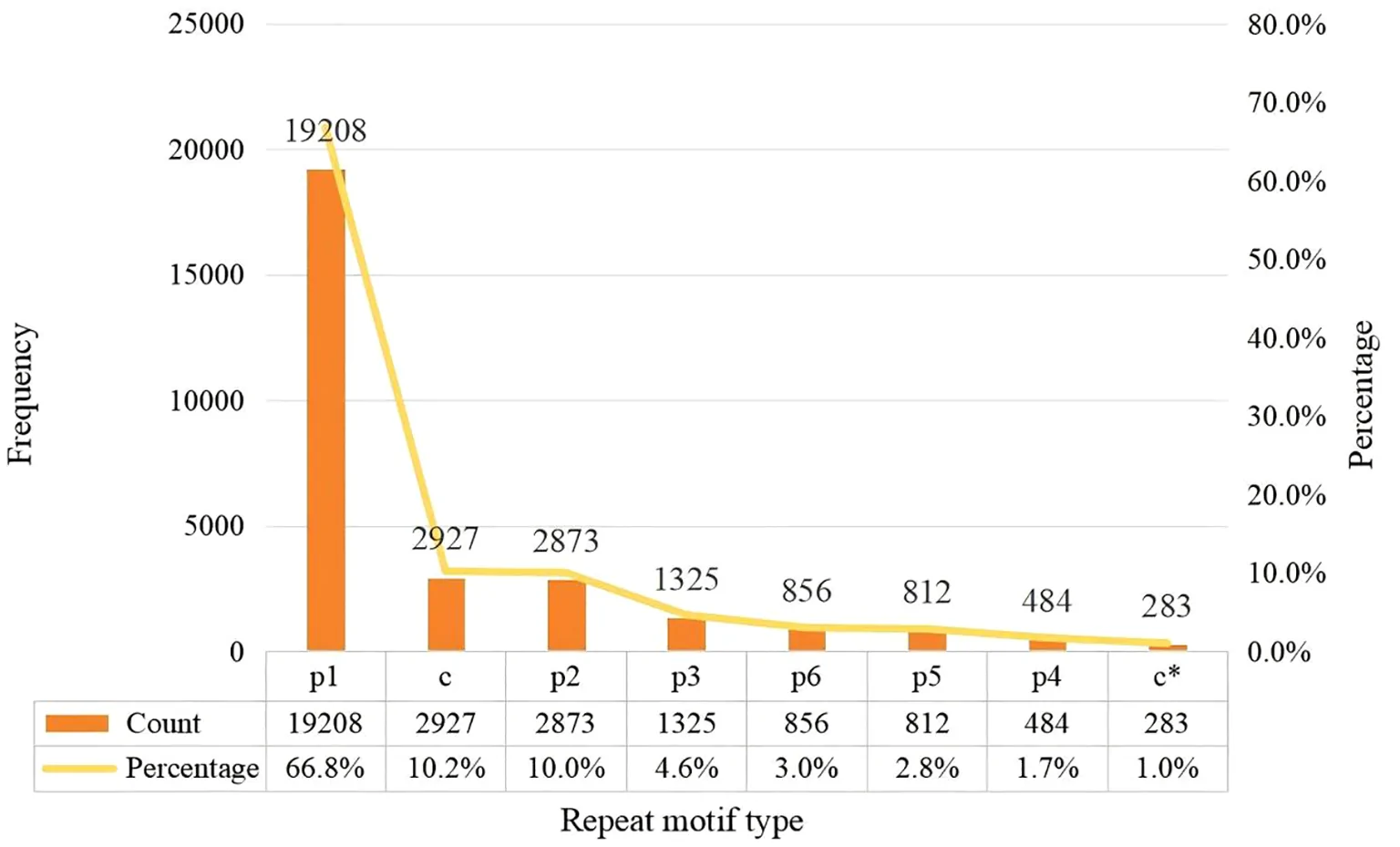
Statistical chart of multi-copy SSR bases with PIC values greater than or equal to 0.5. p1 - p6: represent repeats of 1 to 6 bases, respectively; c: compound SSR marker; c*: imperfect SSR marker.

SSR primers located in multi-copy genes are defined as SSR markers whose flanking sequences have multiple homologous copies in the genome. Such markers are typically located in repetitive sequence regions, and their primers may amplify multiple loci simultaneously. Using the resequencing data of 123 sugar beet samples and the sugar beet genome sequence, we designed 103, 596 SSR primer pairs located on chromosomes and screened 28, 768 SSR primers located in multi-copy genes with a PIC value ≥ 0.5. Among these, 19, 208 had mononucleotide repeats with a minimum of 7 repeats, and 9, 560 had di-or higher-order nucleotide repeats (≥2 bp). For different types of SSR repeat units, the distribution frequency of longer repeat units (penta- and hexanucleotides) decreased significantly as the repeat number increased (**Figure 2**).

**Figure 3:**
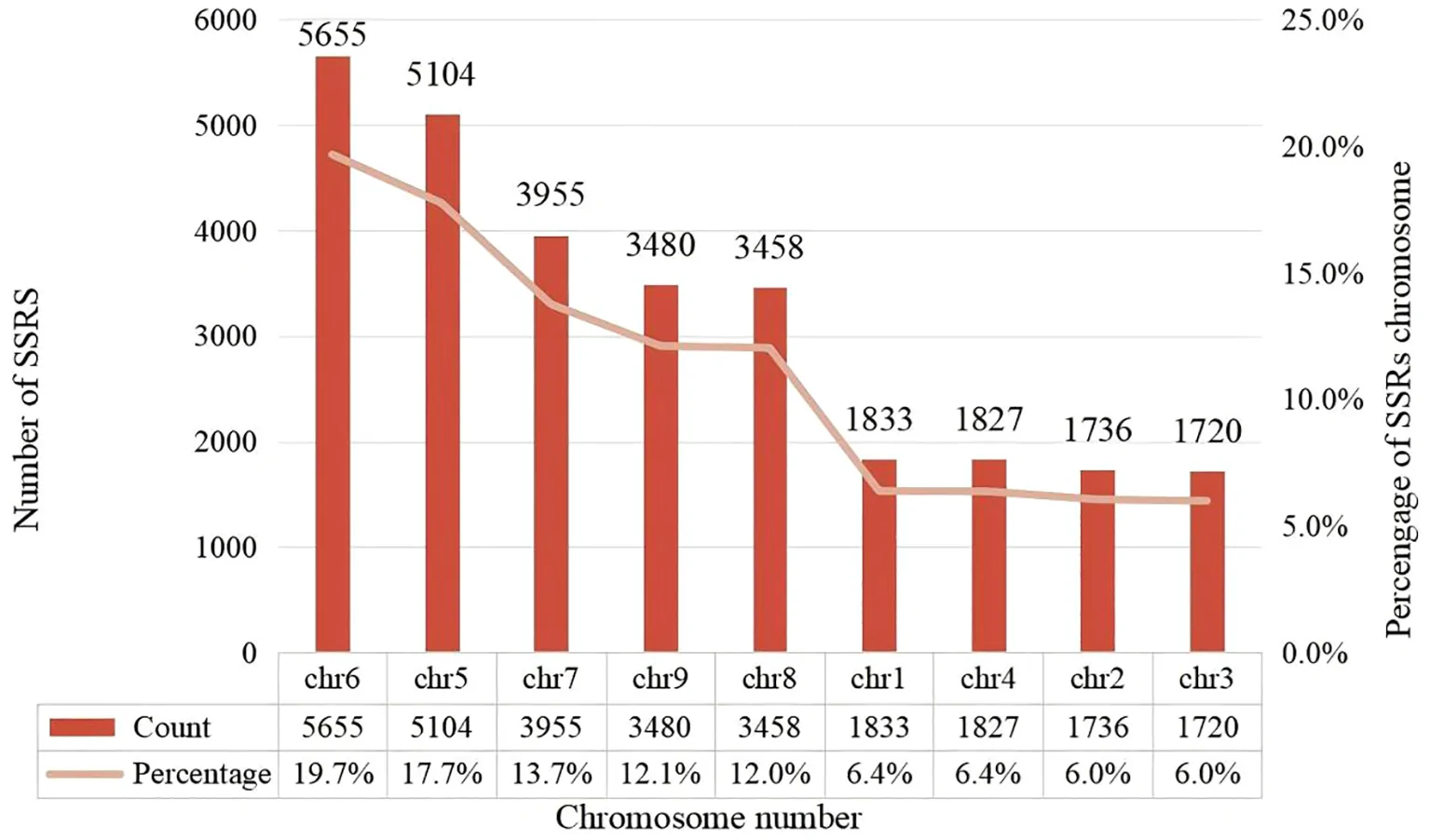
Distribution of multi-copy SSRs with PIC values greater than or equal to 0.5 on 9 chromosomes. chr1 - 9: Represent chromosomes 1 to 9, respectively.

After analysing the repeat types of SSR primers located in multi-copy genes, we further examined the chromosomal distribution of SSR primers located in multi-copy genes with PIC ≥ 0.5 across the nine chromosomes of sugar beet. The PIC value reflects the ability of an SSR primer to distinguish populations. A PIC value > 0.5 indicates a highly polymorphic SSR marker, a value between 0.25 and 0.5 indicates moderately polymorphic, and a value < 0.25 indicates low polymorphism. The distribution of SSR primers located in multi-copy genes with PIC ≥ 0.5 showed significant differences among chromosomes (**Figure 3**). Chromosomes 6, 5 and 7 together accounted for 51.1% of the highly polymorphic SSR markers (19.7%, 17.7% and 13.7%, respectively), with chr6 ranking first with 5, 655 markers (19.7%). Chromosomes 8 and 9 accounted for 12.1% and 12%, respectively, with 3, 458 and 3, 480 markers, both around 3, 500. In contrast, chromosomes 1–4 had markedly lower numbers, each containing approximately 1, 800 markers (6.0% – 6.4% each), which is only one third of that of chr6. These results indicate that highly polymorphic SSR markers are not randomly distributed in the sugar beet genome but are highly enriched on specific chromosomes.

### 3.5 Screening of SSR markers located in single-copy genes and their positional analysis.

**Figure 4:**
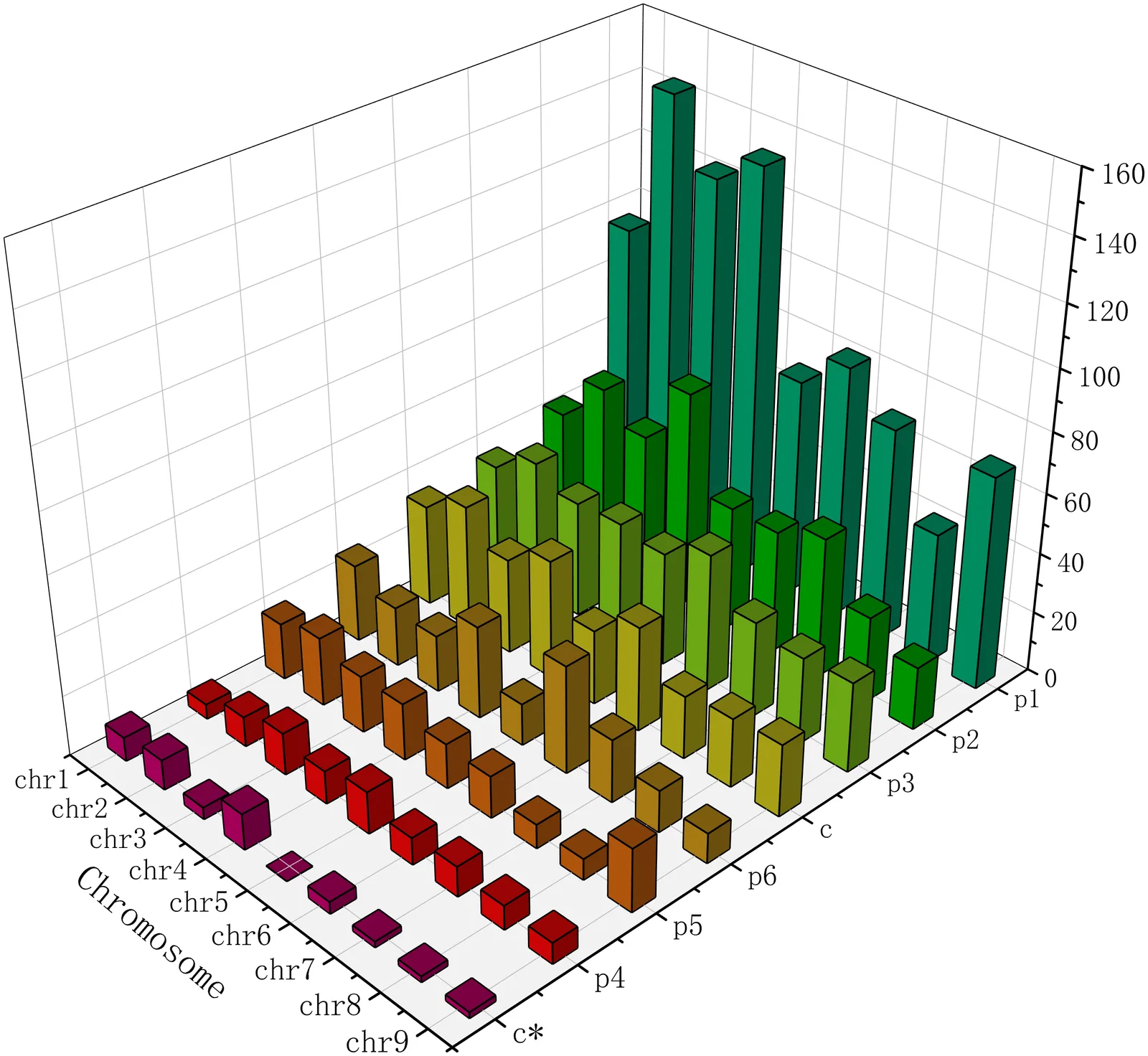
Distribution of repeat types of single-copy SSRs in the 9 chromosomes of sugar beet. p1 - p6: represent repeats of 1 to 6 bases, respectively; c: compound SSR marker; c*: imperfect SSR marker.

A total of 2, 326 SSRs located in single-copy genes were identified in this study. Compared with the 135, 344 SSR markers for which primers were successfully designed, the number of SSR markers located in single-copy gene regions represented only approximately 1.72% of the total. Among them, single nucleotide repeat SSR accounted for the highest proportion (844, 36.3%), followed by di-nucleotide repeat (403, 17.3%) and tri-nucleotide repeat (335, 14.4%). The numbers of tetra-nucleotide, penta-nucleotide, and hexa-nucleotide repeats were 88, 145, and 191 respectively, totaling 424 (18.2%). There were also 276 incomplete SSRs (11.9%) and 44 compound SSRs (1.9%) (**Figure 4**).

For most SSR markers located in single-copy genes, only two genotypes were detected across the 123 varieties, accounting for 910 markers. The next most abundant category was markers with three genotypes (728 markers), followed by those with 4–6 genotypes (606 markers). Markers with one genotype numbered 64, those with seven genotypes numbered 13, those with eight genotypes numbered 3, and those with nine genotypes numbered 2.

**Figure 5:**
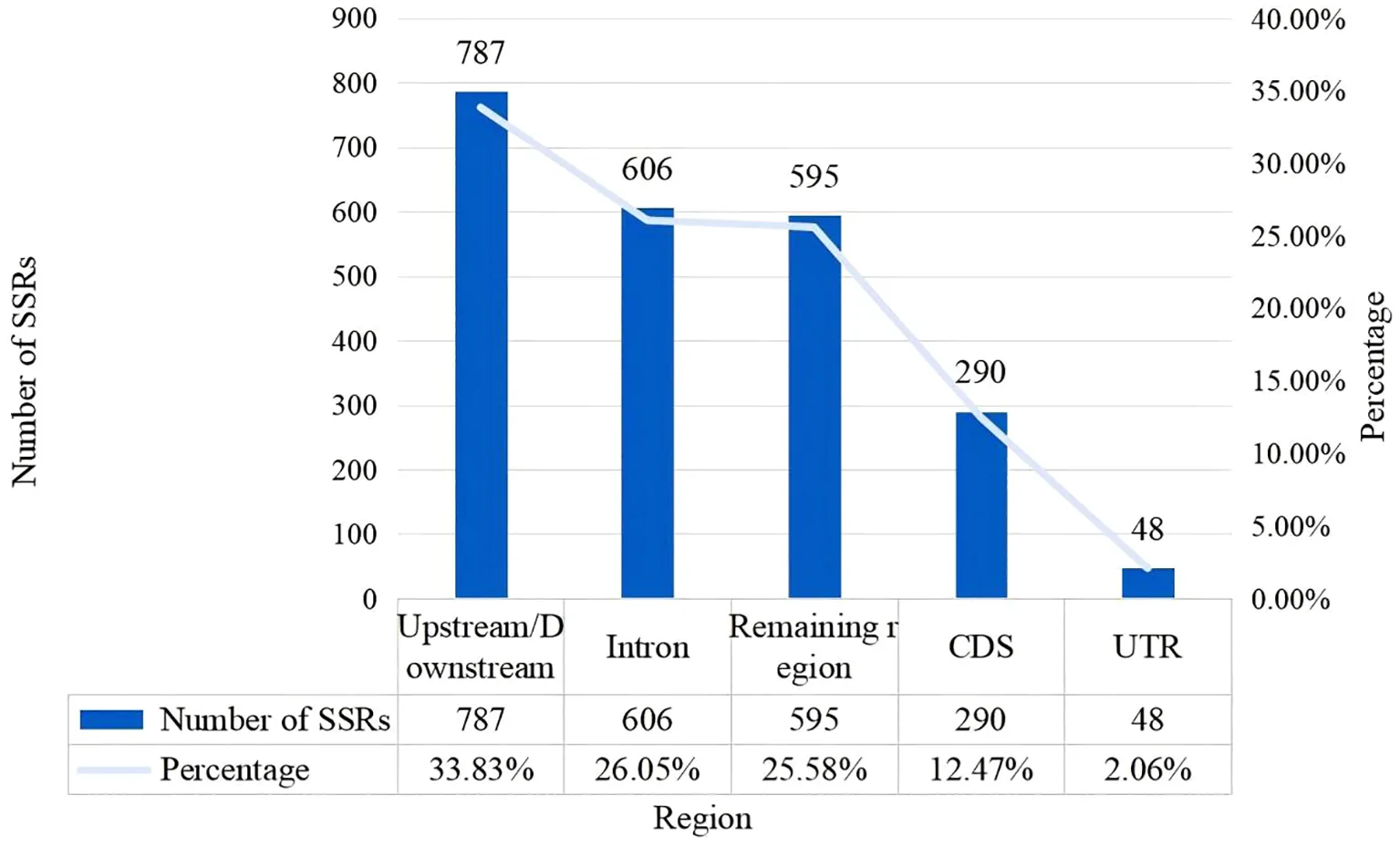
Location of single-copy SSRs in genes. Upstream/Downstream: Gene upstream and downstream regions; Intron: Intron; CDS: Coding region; UTR: Untranslated region; Remaining region: Remaining region.

Analysis of the positions of these SSR markers within genes (**Figure 5**) revealed that, of the 2, 326 SSRs located in single-copy genes, 290 were located entirely in coding regions, 606 in introns, 787 predominantly in upstream/downstream regions, 48 in untranslated regions (UTRs), and the remaining 595 spanned two regions. Owing to their locus specificity, these 2, 326 SSRs located in single-copy genes are more reliable than multi-copy markers for genetic mapping and marker-assisted selection.

### 3.6 Chromosomal distribution and polymorphism analysis of SSR markers located in single-copy genes

The results showed that a total of 2, 326 SSR markers located in single-copy genes were obtained in this study. These markers exhibited an uneven distribution across the nine sugar beet chromosomes, with numbers ranging from 156 (chr8) to 360 (chr4). Among them, chr4 had the highest number (360), followed by chr2 (355) and chr3 (302); chr8 had the lowest number (156), with chr7 and chr9 having intermediate numbers of 212 and 186, respectively.

**Figure 6:**
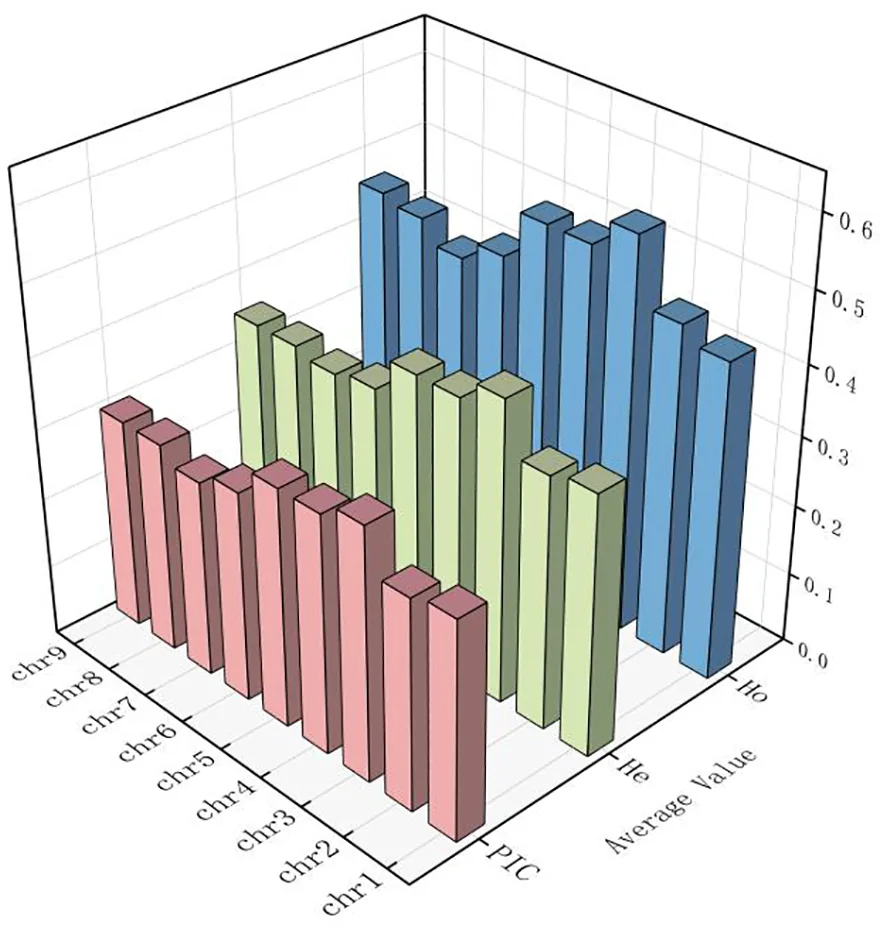
Polymorphism Analysis of Single-Copy SSR Loci. Chromosomes 1-9: Chromosomes 1 to 9.

The average values of observed heterozygosity (Ho), expected heterozygosity (He) and polymorphism information content (PIC) for SSR markers located in single-copy genes on the nine chromosomes were calculated using Cervus software, and the results are shown in **Figure 6**. Overall, levels of genetic diversity varied among chromosomes. Chromosome 3 showed the highest values for Ho, He and PIC (0.56, 0.43 and 0.36, respectively), whereas chromosome 7 showed the lowest (0.42, 0.34 and 0.28, respectively). Notably, chromosomes 4 and 5 had identical parameters (Ho = 0.52, He = 0.40, PIC = 0.34), suggesting that these two chromosomes may share a similar genetic structure. The remaining chromosomes (1, 2, 6, 8 and 9) had relatively similar parameters, with Ho ranging from 0.45 to 0.47, He from 0.35 to 0.37, and PIC from 0.30 to 0.31. These results indicate that the polymorphism information content of SSRs located in single-copy genes on all nine chromosomes reached a moderate level, providing effective markers for future genetic map construction, association analysis and germplasm evaluation.

### 3.7 Functional annotation and pathway enrichment analysis of genes harboring SSRs located in single-copy genes

GO (Gene Ontology) enrichment analysis encompassed three ontologies—Biological Process (BP), Molecular Function (MF), and Cellular Component (CC)—enabling us to identify significantly enriched terms that reflect the functional insights of gene activities. KEGG (Kyoto Encyclopedia of Genes and Genomes) pathway analysis was employed to identify enriched metabolic and signaling pathways, thereby providing insights into the broader biological functions associated with our gene set. To explore the biological significance of SSRs located in single-copy genes, we performed a comprehensive functional annotation of the corresponding genes by integrating information from the NR (Non-Redundant Protein Sequence Database), UniProt (Universal Protein Resource), KEGG, GO, Pfam(Protein families Database Enrichment Analysis), and eggNOG(evolutionary genealogy of genes: Non-supervised Orthologous Groups Enrichment Analysis) databases. Of the 2, 326 SSRs located in single-copy genes identified, a total of 1, 264 (54.3%) were successfully localized to 967 sugar beet genes with well-defined functional descriptions (**Supplementary Table 5**). The functional annotation of gene transcripts derived from the GO database revealed that these genes participate in critical biological processes during sugar beet development and adaptation. Notably, a substantial proportion of the annotated genes were associated with carbohydrate metabolism. Among them, gene-LOC104888538 encodes a protein involved in carbohydrate phosphatase activity, and gene-LOC104889154 encodes a pyruvate dehydrogenase kinase that participates in the regulation of glucose metabolism. Another major category encompassed stress response and defense, including gene-LOC104891113, which is involved in molybdopterin cofactor binding and heme binding, and gene-LOC104891197, which is associated with the plant-pathogen interaction pathway. Genes involved in protein ubiquitination (gene-LOC104887451) and signal transduction (gene-LOC104897766, encoding a NIMA-related kinase) were also prominently distributed.

### 3.8 Validation of SSR primer effectiveness and polymorphism analysis

To evaluate the effectiveness of the developed SSR markers, 48 SSR primer pairs were selected following the criteria described in Section 2.2.7. An initial screening was conducted using DNA from eight sugar beet varieties as templates, with amplification products resolved by 8% non-denaturing polyacrylamide gel electrophoresis. Among the 24 markers located in multi-copy genes, 14 exhibited only 1–2 polymorphic patterns, whereas 10 SSR markers located in single-copy genes displayed 2–3 polymorphic patterns. From this initial screen, 20 primer pairs producing clear, reproducible bands and polymorphic patterns were retained and subsequently evaluated across an expanded panel of 48 varieties. At the population scale, the multi-copy gene-associated marker YCD-4–2 displayed the highest polymorphism, detecting 11 distinct patterns across the 48 varieties, while the single-copy gene-associated marker YCS-7–5 detected 8 patterns. The electrophoresis results showed clear bands without significant smearing, indicating good performance and high resolution. Although the SSR markers located in single-copy genes exhibited lower polymorphism than those in multi-copy genes, their gene-associated locations and unambiguous chromosomal addresses render them particularly suitable for QTL mapping and marker-assisted selection.

The discrepancy between the computationally predicted and experimentally observed PIC values for SSRs located in single-copy genes can be attributed to an interplay of sampling, biological, and technical factors. First, the initial PIC calculation leveraged resequencing data from 123 genetically diverse varieties, whereas wet-lab validation was constrained to an 8-variety panel. This significant reduction in population size and genetic breadth inevitably leads to the non-detection of rare alleles, thereby deflating observed diversity estimates—a well-recognized consequence of the population-specific nature of PIC (Serrote et al., 2020). Second, SSRs located in single-copy genes are predominantly located within or adjacent to genic regions, which are subject to stronger purifying selection than intergenic loci located in multi-copy genes. This selective constraint inherently limits the upper bound of allelic diversity at these sites, restricting most markers to only 2–3 polymorphic types and resulting in lower PIC values compared to their counterparts located in multi-copy genes. This observation aligns with findings in sugarcane, where genic SSRs consistently exhibit lower mean PIC values than genomic SSRs (Kushwah et al., 2020).Third, the technical resolution of 8% non-denaturing polyacrylamide gel electrophoresis (approximately 5–10 bp) is substantially lower than that of HipSTR-based genotyping from sequencing data. This limited resolution can merge alleles of similar lengths, causing further underestimation of polymorphism. Collectively, these factors explain the systematic downward shift of PIC values in wet-lab validation.

The amplification results of the 20 SSR markers on the eight sugar beet varieties are shown in **Table 4**. YCD-5–1 and YCD-7 exhibited the highest number of polymorphic patterns (eight). The results of genetic diversity analysis are presented in **Table 5**.

**Table 4:** SSR marker sequence and amplification results YCS, markers for single-copy SSR; YCD, markers for multi-copy SSR.

Chromosome	Marker	Forward primer(5'-3')	Reverse primer(5'-3')	Polymorphism
1	YCS-1-8	GGCCAAACTTGTACCAAACACA	TGGATTGACTCGATGGACAAGT	2
2	YCS-2-1	TTTCCTTGCCCCAAAGTGCT	TGCAGTCAAGCAAACCACAC	3
4	YCS-4-1	TGCCTCGTATCCTCCTCCAA	GGAGCTGATCTTGAGTGGGG	3
5	YCS-5-1	ACCGTAGAAACAAGGCAAACC	TGGGTTATTAGGGCAACTGGG	3
6	YCS-6-1	TGGGCACGTGTATATGTTCCA	ACGCAGTTGAGCCTACACAA	3
7	YCS-7-1	CCAAAACCTTGAGCTTGGCC	TCCACCTCCTCCTTTCATTCC	2
8	YCS-8-1	GGCACCTTTTTGTCTTCATGA	ACGTCAGATACTTACACTGGCC	3
8	YCS-8-2	GAGGGAGAAGAGCCAAGCAA	GGGGTCTTGCCTGAATCTTCA	3
8	YCS-8-4	AAGAACCAAGCAACAGAACT	AGTTCCATTTGCAGGTTCTT	2
9	YCS-9-1	TCCCAGAACCCTGCATTAGC	GTTGGGGGTACATTTTGGCG	2
2	YCD-2-1	ACAAATGATTGCCTCTTGAATTCA	ACCCTATGCATAACAAAATCCA	7
3	YCD-3-1	TGGGAATTGTGGTAAATGTTACAAGT	ACACACTCCATTTATCTCTCTTGCT	6
4	YCD-4-1	TCTGTGAAAGTTCCTCAGATCTC	TTTGCGGATTGATTTTAGCG	4
4	YCD-4-2	ACTGAAAGAGATGCAGGGTGG	CCAGTTGGTTTTGGTACTTTGGA	5
5	YCD-5-1	TTGCTGCTCCACCTCAACAA	AGGAACGGCGAATTCCAAGT	8
6	YCD-6-1	GGTGGTTGTTGTTGTTGGGG	TCTCTCCTCCAACCACCACT	5
6	YCD-6-2	TCTGCTGATGAGGGGAAGGA	GGGGCATTTTCCATTCTCGG	5
7	YCD-7-1	ACGTGGCCCTCTTCCTATCT	TGACAACACCACCATCACCA	8
8	YCD-8-1	CGTTGAAGTGGGGCGAAGTA	ACAACCCGACTTTCGCTTCT	4
9	YCD-9-1	CGAGAAAAGGCCGAATGTTGA	TGATTTTGTTTTCTGTCTGTTCA	5

**Table 5:** Genetic diversity analysis was performed on eight sugar beet varieties using 20 pairs of primers.

Chromosome	Marker	Ho	He	PIC	Ae	Ar	I
1	YCS1-8	1	0.37	0.34	1.588	3	0.684
2	YCS2-1	1	0.32	0.269	1.471	2	0.5
4	YCS4-1	0.375	0.305	0.258	1.438	2	0.483
5	YCS5-1	1	0.583	0.53	2.4	4	1.075
6	YCS6-1	1	0.46	0.41	1.852	3	0.802
7	YCS7-1	1	0.667	0.593	3	3	1.099
8	YCS8-1	0.625	0.43	0.337	1.753	2	0.621
8	YCS8-2	1	0.49	0.37	1.96	2	0.683
8	YCS8-4	1	0.42	0.332	1.724	2	0.611
9	YCS9-1	1	0.494	0.438	1.976	3	0.849
2	YCD2-1	1	0.721	0.67	3.585	4	1.326
3	YCD3-1	1	0.796	0.765	4.9	6	1.66
4	YCD4-1	1	0.73	0.686	3.706	5	1.427
4	YCD4-2	1	0.704	0.657	3.379	5	1.376
5	YCD5-1	1	0.826	0.802	5.753	6	1.77
6	YCD6-1	1	0.762	0.726	4.195	6	1.59
6	YCD6-2	1	0.749	0.706	3.981	5	1.468
7	YCD7-1	1	0.832	0.808	5.939	6	1.787
8	YCD8-1	1	0.73	0.68	3.704	4	1.345
9	YCD9-1	1	0.773	0.735	4.404	5	1.531

YCS, markers for single-copy SSR; YCD, markers for multi-copy SSR.

Ho, observed heterozygosity;He, expected heterozygosity;PIC, polymorphism information content; Ae, Effective Number of Alleles; Ar, Allelic Richness;I:Shannon's Information Index.

**Figure 7:**
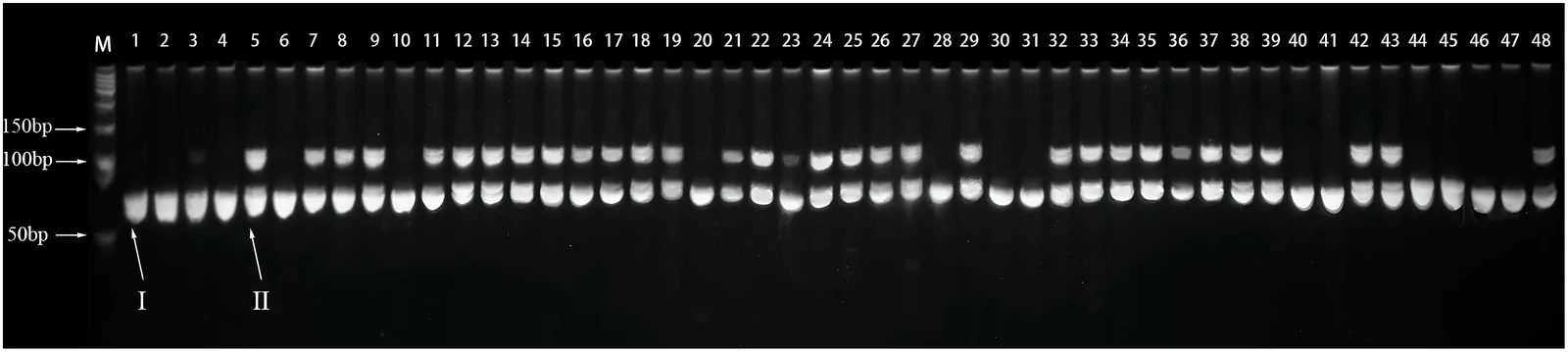
Amplification profiles of marker YCS-4-1 on 48 sugar beet samples. Arabic numerals, beet variety numbers; Roman numerals, different polymorphic patterns; M, DNA molecular weight marker (50 bp ladder).

**Figure 8:**
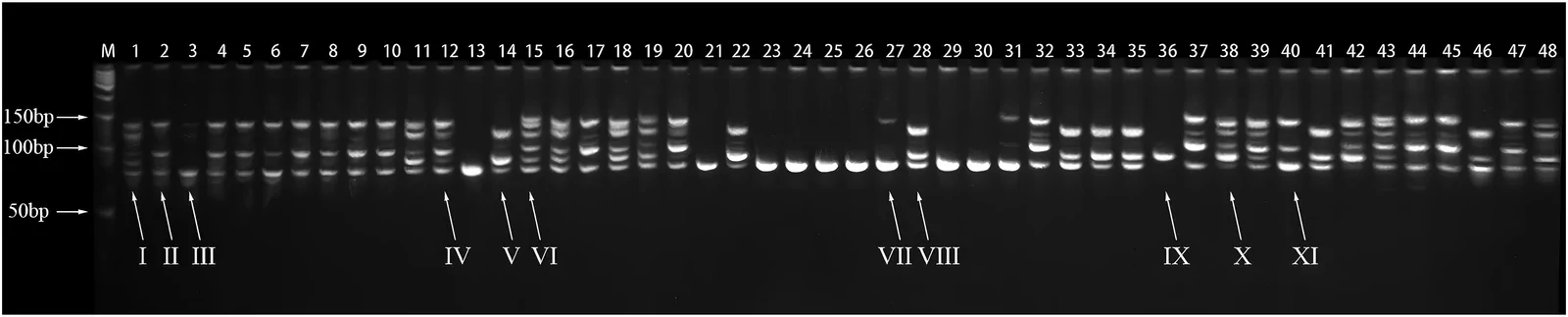
Amplification profiles of marker YCD-4-2 on 48 sugar beet samples. Arabic numerals, beet variety numbers; Roman numerals, different polymorphic patterns; M, DNA molecular weight marker (50 bp ladder).

To further assess the quality of these primers, we selected the SSR marker located in a single-copy gene YCS-7–5 and the SSR marker located in multi-copy genes YCD-4–2 to analyse their polymorphism levels across 48 sugar beet varieties using 8% non-denaturing polyacrylamide gel electrophoresis (**Figures 7**, **8**). By comparing band sizes, YCD-4–2 detected 11 polymorphic patterns among the 48 varieties, whereas YCS-7–5 detected 8 polymorphic patterns. The electrophoresis results showed clear bands without significant smearing, indicating good performance and high resolution. Among the initial 8-variety screen, YCD-5–1 and YCD-7 showed the greatest polymorphism (8 patterns). Extending YCD-4–2 across 48 varieties revealed 11 patterns, underscoring that polymorphism assessment is sensitive to the number of varieties screened. The variety number corresponding to each lane in **Figures 7**, **8** is provided in **Supplementary Table 3**.

## 4 Discussion

Compared with the traditional approach of developing SSR markers based on transcriptome data, whole-genome resequencing enables genome-wide coverage of both genic and non-coding regions, circumventing the limitations of transcriptome data (Zhang et al., 2012; Xia et al., 2018; Chapman, 2019). Wang et al. (Wang Y. et al., 2023) conducted a study that clearly demonstrated that due to the limitations of read length and assembly quality of transcriptome data, the EST-SSR markers developed from single transcriptomes often lack sufficient validation and have low polymorphism. The polymorphism in their study was only 29%.Similarly, Ding et al. (Ding et al., 2015) characterized EST-SSRs in Chinese cabbage using deep transcriptome sequencing, reporting low polymorphism and limited marker development efficiency. Tian et al. (Tian et al., 2022) identified 97, 510 SSR loci based on full-length transcriptome data, but their validation showed an amplification efficiency of only 51.72% for SSR primers and a development efficiency of only 26.72% for polymorphic SSR primers.

The functional annotation of genes harboring SSR markers located in single-copy genes revealed a significant enrichment in two biological processes—carbohydrate metabolism and stress response—that have defined sugar beet breeding objectives for the past two centuries (Çelik, 2023). Among the 967 annotated genes, several encode key enzymes directly involved in sugar metabolism, including a pyruvate dehydrogenase kinase (LOC104889154) that regulates glucose metabolic flux, and carbohydrate phosphatase-encoding genes that participate in sugar interconversion pathways. The localization of SSR loci within or immediately adjacent to these genes raises the possibility that allelic variation at these markers may be associated with differences in sugar accumulation phenotypes. This hypothesis is supported by the presence of SSRs in sucrose transporter (SUT) family members, whose expression levels have been correlated with sucrose content in sugar beet taproots in independent studies (Dhiman et al., 2025). Similarly, the enrichment of SSR markers located in single-copy genes in genes implicated in plant-pathogen interactions, including those encoding NB-LRR resistance proteins and molybdopterin cofactor-binding enzymes, is of parallel interest.

Both KEGG and GO enrichment analyses consistently indicated that the genes located within single-copy genes that contained SSRs were significantly enriched in the pathways of carbohydrate metabolism (KEGG: enrichment fold = 1.41, FDR = 6.30×10^-5^; GO: enrichment fold = 1.37, FDR = 1.58×10^-5^) and stress response (KEGG: enrichment fold = 1.25, FDR = 5.58×10^-3^; GO: enrichment fold = 1.33, FDR = 1.97×10^-6^). The enrichment of the carbohydrate metabolism pathway is particularly noteworthy, as sugar crops like sugar beets, which accumulate sucrose as the core of their sugar content, involve a large number of key enzymes and transport proteins in the synthesis, transport, and storage of sugar in their root tissues. The presence of SSRs within or near these genes suggests that these markers may be directly associated with sugar accumulation traits. The SSR markers related to NB-LRR resistance proteins and molybdenum cofactor binding enzymes identified in the stress response pathway have the potential to serve as functional markers for disease screening. Although the plant-pathogen interaction pathway showed marginal enrichment (KEGG: 1.06 times, FDR = 0.125; GO: 1.11 times, FDR = 0.303), combined with the identification results of the above disease-related genes, the application value of these markers in disease breeding still deserves further exploration. In summary, the consistent results of the two independent enrichment analyses further support the functional correlation of these gene-related SSR markers in sugar metabolism regulation and stress response.

If similar regulatory mechanisms operate in sugar beet, the SSR markers identified in these defense-related genes could serve as gene-associated SSRs for disease resistance screening. Taken together, these functional annotations extend the 2, 326 SSR markers located in single-copy genes beyond a catalog of polymorphic loci to a targeted resource for dissecting the genetic architecture of sugar content and disease resistance. We propose that the 543 SSR markers located in single-copy genes with PIC ≥ 0.5 that are located in or near functionally annotated genes should be prioritized in future association mapping studies aimed at identifying quantitative trait loci for sugar yield and disease resistance. This strategy is supported by previous successes in sugar beet, where SSR markers have been effectively used to dissect the genetic basis of complex traits such as sugar content and yield, and in other crops (Çelik, 2023), where gene-associated SSRs have proven valuable for linking molecular variation to phenotypic traits (Choudhary et al., 2021; Hemasai et al., 2024; Hou et al., 2026).

Based on whole-genome resequencing of 123 sugar beet varieties, this study not only developed SSR primers located in multi-copy genes but also screened SSR markers located in single-copy genes. Transcriptome data, owing to the lack of complete genome structural information, make it difficult to accurately determine the copy number of SSR flanking sequences in the genome, often leading to the misidentification of multi-copy sequences as candidate markers. The available sugar beet (*Beta vulgaris* L.) genome sequence shows that repetitive sequences account for approximately 60 – 70% of the entire genome; therefore, distinguishing SSR markers located in single-copy genes from those in multi-copy genes is particularly critical for precise genetic localization. The primary goal of SSR marker development is to identify single-copy sequences containing SSR loci, because only SSR sequences located in single-copy genes can be accurately mapped to a unique chromosomal locus.

Previous reports have described SSR marker development in sugar beet. Dohm et al. (Dohm et al., 2014) initially developed some SSR markers based on the reference genome but did not perform large-scale polymorphism screening. Çelik (Çelik, 2023) developed 22, 500 SSR primer pairs using genome-wide data but did not further distinguish SSR markers located in single-copy genes from those located in multi-copy genes, nor did they provide the number of highly polymorphic markers with PIC ≥ 0.5. The contributions of our study are as follows: we increased the number of available primer pairs to over 130, 000; we systematically screened, for the first time in sugar beet, SSR markers located in single-copy genes (2, 326); and we experimentally validated 20 reliable markers with defined polymorphism levels. This validation step is of critical importance for breeders. Among the 28, 768 SSR primers located in multi-copy genes with PIC ≥ 0.5, 2, 873 were dinucleotide repeats, representing the most abundant type, whereas the numbers of tetra-, penta- and hexanucleotide repeats (with a minimum of 3 repeats) were far lower than that of dinucleotide repeats. This distribution pattern is consistent with the SSR distribution observed by Tóth et al. (Tóth et al., 2000) across different eukaryotic genomes, where dinucleotide repeats dominate in introns and intergenic regions, further supporting the biological rationality of our results.

This study found that SSR markers located in multi-copy genes were most abundant on chr6, whereas they were relatively scarce on chr1 – 4. This distribution pattern may be associated with the evolutionary history of the sugar beet genome. Sugar beet has experienced a paleotriploidy event and retains a large proportion of repetitive sequences in its genome (accounting for approximately 60 – 70%). We hypothesize that the enrichment of SSR markers located in multi-copy genes on chr6 may be associated with its greater physical length and potentially elevated transposon density; however, this requires investigation using repeat landscape analysis. Notably, although chr3 harbors the fewest SSR primers (8, 067) among all chromosomes, it exhibits the highest marker frequency (303.6 per Mb). This pattern reflects its relatively small physical size (26.58 Mb), which results in a higher marker density per unit length despite the low absolute count. The higher genetic diversity observed on chromosome 3 (Ho = 0.56) relative to chromosome 7 (Ho = 0.42) is consistent with previously reported relationships between chromosomal features and recombination rates. In large-genome plant species, recombination is typically suppressed in pericentromeric regions due to the enrichment of repetitive DNA, whereas gene-rich euchromatic regions and more distally located centromeres are associated with higher recombination frequencies (Zelkowski et al., 2019). In contrast, SSRs located in single-copy genes were most abundant on chr4, suggesting that this chromosome may have retained more evolutionarily conserved low-copy regions, making it suitable for developing gene-associated SSRs. This distribution feature provides a reference for chromosome prioritization in subsequent marker-assisted selection.

The primer sequences of the 20 validated SSR markers, along with the figures and tables, are provided in the **Supplementary Materials**. The sugar beet reference genome (RefBeet-1.2.2) used in this study is publicly accessible through the NCBI database. The raw resequencing data of the 123 sugar beet varieties will be made publicly available upon acceptance.The eight varieties used for initial screening were strategically selected to encompass all six breeding companies represented in the full collection of 123 accessions—namely, KWS SAAT SE, Strube, Lion Seeds Ltd., SES VanderHave, MariboHilleshög Aps, and BETASEED. This design ensures that the screening panel captures the maximal breadth of commercial breeding lineages within the studied germplasm. If a primer pair yields two or more distinct band patterns across these eight varieties, it provides experimental confirmation that the marker is polymorphic and, concurrently, validates the effectiveness of the bioinformatic selection criterion—PIC ≥ 0.5 predicted from resequencing data—as described in Section 2.2.7. The eight-variety screen therefore serves an experimental validation function, the purpose of which is to confirm that the bioinformatic selection strategy effectively identifies polymorphic markers, rather than to provide a comprehensive assessment of diversity across the full germplasm. The subsequent evaluation of all 20 validated markers across 48 varieties further substantiates their polymorphic potential and demonstrates that the initial screening approach did not introduce systematic bias. This phased validation strategy employs initial screening on a representative subset followed by expanded testing across a larger population. This approach balances experimental efficiency with analytical rigor and is consistent with established practices in large-scale SSR marker development.

In practical breeding, SSR markers located in multi-copy genes and SSR markers located in single-copy genes possess complementary value, and the two types of markers should be selected differentially according to the target traits and application scenarios. SSR markers located in multi-copy genes are abundant and highly polymorphic, enabling variety discrimination with a small number of markers, and can be preferentially used in scenarios such as germplasm resource identification, genetic diversity analysis, and hybrid seed purity testing, thereby reducing genotyping costs. SSRs located in single-copy genes, in contrast, owing to their strong locus specificity and freedom from paralogous interference, are better suited for applications that require high mapping precision, such as fine QTL mapping and gene function studies. Moreover, SSRs located in single-copy genes exhibit superior amplification stability, offering advantages in cross-laboratory validation and long-term application. The 2, 326 SSR markers located in single-copy genes provided in this study can be directly applied to such precision breeding efforts.

The marker selection strategy employed in this study—prioritizing loci based on PIC ≥ 0.5, predicted allele number, and detection rate—represents a systematic, criteria-based approach informed by resequencing data. Future studies could further enhance this strategy by incorporating predictive tools such as the MultiplexSSR pipeline (Guo et al., 2020), which uses resequencing data from a small number of individuals to predict allele numbers and length ranges in silico prior to primer synthesis, thereby enabling more efficient prioritization of highly polymorphic loci for validation.

Despite the above progress, this study has limitations. First, the average sequencing depth of 123 sugar beet varieties was approximately 10×, which limits sensitivity for detecting rare variants and small indels within primer binding sites, potentially underestimating true genetic diversity. Second, wet-lab validation based on 8% non-denaturing PAGE involved only eight varieties. Although representing major breeding lineages, this sample size may not fully capture fragment length polymorphism across broader germplasm. Third, all accessions were cultivated beet (*Beta vulgaris*); the transferability and polymorphism of the 2, 326 SSRs located in single-copy genes have not been tested in wild relatives (e.g., sea beet Beta vulgaris ssp. maritima). Given the much higher genetic diversity of wild populations, cross-population validation is indispensable before applying these markers to introgressive breeding or germplasm conservation. Finally, SSRs located in single-copy genes account for only 1.72% of primer-designable SSR markers in the sugar beet genome, indicating limited genomic coverage. A methodological note is warranted: the classification of SSR markers in this study is based on gene copy number at the protein level, not on SSR locus copy number at the DNA level. Consequently, confirmation of single-locus amplification would require additional experimental validation.

## 5 Conclusion

Based on resequencing data of 123 sugar beet varieties, we developed a genome-wide SSR marker resource comprising 135, 344 primer pairs, including 28, 768 SSR markers located in multi-copy genes with high polymorphism (PIC ≥ 0.5) and 2, 326 SSR markers located in single-copy genes. Following experimental validation, 20 practical markers with clear bands, good reproducibility and polymorphism were obtained, among which the most polymorphic marker, YCD-4-2, detected 11 polymorphic patterns across 48 varieties. The 543 SSRs located in single-copy genes with PIC ≥ 0.5 represent priority candidates for QTL mapping and association analyses; the remaining markers may also provide value in low-diversity populations.

## Data Availability

The data presented in the study are deposited in the Sequence Read Archive (SRA)A repository, accession number PRJNA1495228.

## References

[B1] Addou S. Rentzsch R. Lee D. Orengo C. A. (2009). Domain-based and family-specific sequence identity thresholds increase the levels of reliable protein function transfer. J. Mol. Biol. 387, 416–430. doi: 10.1016/j.jmb.2008.12.045 19135455

[B2] Buchfink B. J. Barbé É. Ashkenazy H. Reuter K. Kennedy J. A. Drost H.-G. (2026). Clustering the protein universe of life using DIAMOND DeepClust. Nat. Methods 23, 724–727. doi: 10.1038/s41592-026-03030-z 41876643 PMC13076203

[B3] Çelik İ. (2023). Genome-wide development and physical mapping of SSR markers in sugar beet (Beta vulgaris L.). J. Inst. Sci. Technol. 13, 112–119. doi: 10.21597/jist.1187003

[B4] Chapman M. A. (2019). Optimizing depth and type of high-throughput sequencing data for microsatellite discovery. Appl. Plant Sci. 7, e11298. doi: 10.1002/aps3.11298 31832281 PMC6858294

[B5] Choudhary S. Naika M. B. N. Meena R. D. (2021). Development and characterization of genic SSR-FDM for stem gall disease resistance in coriander (Coriandrum sativum L.) and its cross species transferability. Mol. Biol. Rep. 48, 3963–3970. doi: 10.1007/s11033-021-06396-9 34021895

[B6] Dhiman A. S. Taliadoros D. Stukenbrock E. H. McGrath J. M. Emrani N. Jung C. (2025). Signatures in domesticated beet genomes pointing at genes under selection in a sucrose-storing root crop. BMC Biol. 23, 299. doi: 10.1186/s12915-025-02422-5 41057828 PMC12505646

[B7] Ding Q. Li J. Wang F. Zhang Y. Li H. Zhang J. . (2015). Characterization and development of EST-SSRs by deep transcriptome sequencing in Chinese cabbage (Brassica rapa L. ssp. pekinensis). Int. J. Genomics 2015, 473028. doi: 10.1155/2015/473028 26504770 PMC4609433

[B8] Dohm J. C. Minoche A. E. Holtgräwe D. Capella-Gutiérrez S. Zakrzewski F. Tafer H. . (2014). The genome of the recently domesticated crop plant sugar beet (Beta vulgaris). Nature 505, 546–549. doi: 10.1038/nature12817 24352233

[B9] Doolittle R. F. (1986). Of Urfs and Orfs: a primer on how to analyze derived amino acid sequences. In University Science Books (Herndon, VA: University Science Books). 29, 1–103. doi: 10.1002/jobm.3620290411

[B10] Felkel S. Dohm J. C. Himmelbauer H. (2023). Genomic variation in the genus Beta based on 656 sequenced beet genomes. Sci. Rep. 13, 8654. doi: 10.1038/s41598-023-35691-7 37244945 PMC10224960

[B11] Geethanjali S. Kadirvel P. Anumalla M. Sadhana N. H. Annamalai A. Ali J. (2024). Streamlining of simple sequence repeat data mining methodologies and pipelines for crop scanning. Plants 13, 2619. doi: 10.3390/plants13182619 39339594 PMC11435353

[B12] Goldman I. L. Janick J. (2021). Evolution of root morphology in table beet: historical and iconographic. Front. Plant Sci. 12, 689926. doi: 10.3389/fpls.2021.689926 34447400 PMC8384405

[B13] Guo L. Yang Q. Yang J.-W. Zhang N. Liu B.-S. Zhu K.-C. . (2020). MultiplexSSR: A pipeline for developing multiplex SSR-PCR assays from resequencing data. Ecol. Evol. 10, 3055–3067. doi: 10.1002/ece3.6121 32211176 PMC7083706

[B14] Habermann B. H. (2016). Oh Brother, Where Art Thou? Finding Orthologs in the Twilight and Midnight Zones of Sequence Similarity. Evol. Biol. 22, 22, 393–419. doi: 10.1007/978-3-319-41324-2_22

[B15] Hemasai B. Kumbha D. K. Modem V. N. Gannavarapu S. K. Bommaka R. R. Mallapuram S. . (2024). Development of miRNA-SSR and target-SSR markers from yield-associated genes and their applicability in the assessment of genetic diversity and association mapping in rice (Oryza sativa L.). Mol. Breed. 44, 30. doi: 10.1007/s11032-024-01462-z 38634111 PMC11018576

[B16] Hou X. Li X. Jiao Y. Guo X. Zhang D. Gao Y. . (2026). Genetic diversity and association analysis between agronomic traits and EST-SSR markers in Chinese chive (Allium tuberosum). Front. Plant Sci. 17, 1785981. doi: 10.3389/fpls.2026.1785981 41948300 PMC13050861

[B17] Jung Y. Han D. (2022). BWA-MEME: BWA-MEM emulated with a machine learning approach. Bioinformatics 38, 2404–2413. doi: 10.1093/bioinformatics/btac137 35253835

[B18] Kushwah R. B. S. Mahendrakar M. D. Jugran A. K. Singh R. K. (2020). Assessing genetic diversity and population structure of sugarcane cultivars, progenitor species and genera using microsatellite (SSR) markers. Gene 753, 144800. doi: 10.1016/j.gene.2020.144800 32454179

[B19] Li J. Song Y. Li S. Pi Z. Wu Z. (2025). Developing Chinese sugar beet core collection: comprehensive analysis based on morphology and molecular markers. Horticulturae 11, 990. doi: 10.3390/horticulturae11080990 30654563

[B20] Mandal L. Verma S. K. Sasmal S. Katara J. (2018). Potential applications of molecular markers in plant. Curr. Trends Biomed. Eng. Biosci. 12, 555844. doi: 10.19080/ctbeb.2018.12.555844

[B21] Meng Y. Zheng C. Li H. Li A. Zhai H. Wang Q. . (2021). Development of a high-density SSR genetic linkage map in sweet potato. Crop J. 9, 1367–1374. doi: 10.1016/j.cj.2021.01.003 38826717

[B22] Panahi B. Jalaly H. M. Hamid R. (2024). Using next-generation sequencing approach for discovery and characterization of plant molecular markers. Curr. Plant Biol. 40, 100412. doi: 10.1016/j.cpb.2024.100412 38826717

[B23] Puligundla P. Mok C. (2021). Valorization of sugar beet pulp through biotechnological approaches: recent developments. Biotechnol. Lett. 43, 1253–1263. doi: 10.1007/s10529-021-03146-6 33978884

[B24] Sandell F. L. Stralis-Pavese N. McGrath J. M. Schulz B. Himmelbauer H. Dohm J. C. (2022). Genomic distances reveal relationships of wild and cultivated beets. Nat. Commun. 13, 2021. doi: 10.1038/s41467-022-29676-9 35440134 PMC9019029

[B25] Serrote C. M. L. Reiniger L. R. S. Silva K. B. Rabaiolli S. M. S. Stefanel C. M. (2020). Determining the polymorphism information content of a molecular marker. Gene 726, 144175. doi: 10.1016/j.gene.2019.144175 31726084

[B26] Shavrukov Y. Hinrichsen P. Watanabe S. (2024). Editorial: Plant genotyping: from traditional markers to modern technologies. Front. Plant Sci. 15, 1419798. doi: 10.3389/fpls.2024.1419798 38745928 PMC11093220

[B27] Tian Q. Huang B. Huang J. Wang B. Dong L. Yin X. . (2022). Microsatellite analysis and polymorphic marker development based on the full-length transcriptome of Camellia chekiangoleosa. Sci. Rep. 12, 18906. doi: 10.1038/s41598-022-23333-3 36344600 PMC9640616

[B28] Tóth G. Gáspári Z. Jurka J. (2000). Microsatellites in different eukaryotic genomes: survey and analysis. Genome Res. 10, 967–981. doi: 10.1101/gr.10.7.967 10899146 PMC310925

[B29] United States Department of Agriculture (2023). Available online at: https://apps.fas.usda.gov/psdonline/app/index.html#/app/home (Accessed September 15, 2023).

[B30] Wang H. Yang K. Huang H. Guo L. He X. (2023). Development of simple sequence repeat markers and genetic diversity evaluation of Mycocentrospora acerina in Yunnan Province, China. J. Fungi 9, 944. doi: 10.3390/jof9090944 37755052 PMC10532959

[B31] Wang Y. Yang T. Wang X. Sun X. Liu H. Wang D. . (2023). Develop a preliminary core germplasm with the novel polymorphism EST-SSRs derived from three transcriptomes of colored calla lily (Zantedeschia hybrida). Front. Plant Sci. 14, 1055881. doi: 10.3389/fpls.2023.1055881 36818854 PMC9933510

[B32] Wang L. Zhang Z. Han P. Liang Y. Zhang H. Fu Z. . (2023). Association analysis of agronomic traits and construction of genetic networks by resequencing of 306 sugar beet (Beta vulgaris L.) lines. Sci. Rep. 13, 15422. doi: 10.1038/s41598-023-42182-2 37723186 PMC10507079

[B33] Willems T. Zielinski D. Yuan J. Gordon A. Gymrek M. Erlich Y. (2017). Genome-wide profiling of heritable and de novo STR variations. Nat. Methods 14, 590–592. doi: 10.1038/nmeth.4267 28436466 PMC5482724

[B34] Xia Y. Luo W. Yuan S. Zheng Y. Zeng X. (2018). Microsatellite development from genome skimming and transcriptome sequencing: comparison of strategies and lessons from frog species. BMC Genomics 19, 886. doi: 10.1186/s12864-018-5329-y 30526480 PMC6286531

[B35] Xue G. Wu W. Fan Y. Ma C. Xiong R. Bai Q. . (2024). Genome-wide identification, evolution, and role of SPL gene family in beet (Beta vulgaris L.) under cold stress. BMC Genomics 25, 101. doi: 10.1186/s12864-024-09995-5 38262939 PMC10804631

[B36] Yolcu S. Alavilli H. Ganesh P. Asif M. Kumar M. Song K. (2022). An insight into the abiotic stress responses of cultivated beets (Beta vulgaris L.). Plants 11, 12. doi: 10.3390/plants11010012 35009016 PMC8747243

[B37] Zelkowski M. Olson M. A. Wang M. Pawlowski W. (2019). Diversity and determinants of meiotic recombination landscapes. Trends Genet. 35, 359–370. doi: 10.1016/j.tig.2019.02.002 30948240

[B38] Zhang H. Wei L. Miao H. Zhang T. Wang C. (2012). Development and validation of genic-SSR markers in sesame by RNA-seq. BMC Genomics 13, 316. doi: 10.1186/1471-2164-13-316 22800194 PMC3428654

