# Correction: Development and validation of whole-genome SSR markers in sugar beet (*Beta vulgaris* L.)

**DOI:** 10.3389/fpls.2026.1965732

**Published:** 2026-08-20

**Authors:** Chengcheng Yang, Shengnan Li, Zhi Pi, Zedong Wu, Yongli Hao

**Affiliations:** 1 College of Modern Agriculture and Ecological Environment, Heilongjiang University, Harbin, Heilongjiang, China; 2 Chifeng Academy of Agricultural and Animal Husbandry Sciences, Chifeng, China

**Keywords:** genome-wide, polymorphic primers, resequencing, simple sequence repeat markers, sugar beet

A correction has been made to section *2.2.9 Data statistics and analysis.* The original sentence stated:

“The genetic diversity indicators were calculated using popgen1.32, including Ho (observed heterozygosity); He (expected heterozygosity); PIC (polymorphism information content); Ae (Effective Number of Alleles); Ar (Allelic Richness); I (Shannon’s Information Index).”

In this sentence, PIC (polymorphism information content) was incorrectly attributed to “popgen1.32”. In fact, the PIC values reported in our study were calculated using “PowerMarker 3.25”, not popgen1.32.

The corrected sentence reads:

“The genetic diversity indicators were calculated using Popgene 1.32, including Ho (observed heterozygosity), He (expected heterozygosity), Ae (effective number of alleles), Ar (allele richness), and I (Shannon’s information index). PIC (polymorphism information content) was calculated using PowerMarker 3.25.”

The original version of this article has been updated.

