# Supplementary material for: Development and validation of whole-genome SSR markers in sugar beet (Beta vulgaris L.)
Source: Front Plant Sci. 2026 Aug 11;17:1873607. doi: 10.3389/fpls.2026.1873607 (PMC13539604; doi:10.3389/fpls.2026.1873607)
Supplement: Supplementary file 1 [file DataSheet8.docx]

**Supplementary Table S3.** Sample numbers corresponding to each lane in the gel images of Figs. 7 and 8.

| Number | Variety Name | Breeding Company |
| --- | --- | --- |
| 1 | KUHN1260 | SES VanderHave |
| 2 | KUHN5160 | SES VanderHave |
| 3 | MK4185 | SES VanderHave |
| 4 | MK4206 | SES VanderHave |
| 5 | MK4245 | SES VanderHave |
| 6 | MK4256 | SES VanderHave |
| 7 | MK4265 | SES VanderHave |
| 8 | MK4269 | SES VanderHave |
| 9 | MK4271 | SES VanderHave |
| 10 | SV2221 | SES VanderHave |
| 11 | SV2426 | SES VanderHave |
| 12 | SV2431 | SES VanderHave |
| 13 | BTS1175 | BETASEED |
| 14 | BTS2730 | BETASEED |
| 15 | BTS7105 | BETASEED |
| 16 | BTS8430 | BETASEED |
| 17 | BTS8935 | BETASEED |
| 18 | SV2892 | SES VanderHave |
| 19 | SX1521 | SES VanderHave |
| 20 | SX1536 | SES VanderHave |
| 21 | SX1539 | SES VanderHave |
| 22 | SX1541 | SES VanderHave |
| 23 | MA2 | MariboHilleshög ApS |
| 24 | MA4 | MariboHilleshög ApS |
| 25 | MA6 | MariboHilleshög ApS |
| 26 | MA8 | MariboHilleshög ApS |
| 27 | MA10 | MariboHilleshög ApS |
| 28 | MA12 | MariboHilleshög ApS |
| 29 | MA14 | MariboHilleshög ApS |
| 30 | MA20 | MariboHilleshög ApS |
| 31 | MA22 | MariboHilleshög ApS |
| 32 | MA24 | MariboHilleshög ApS |
| 33 | ST12125 | Strube |
| 34 | ST12655 | Strube |
| 35 | ST12803 | Strube |
| 36 | ST12909 | Strube |
| 37 | ST13103 | Strube |
| 38 | ST137118 | Strube |
| 39 | ST13832 | Strube |
| 40 | ST13915_1 | Strube |
| 41 | ST15108 | Strube |
| 42 | L2305 | Lion Seeds Ltd. |
| 43 | L2308 | Lion Seeds Ltd. |
| 44 | LS2301 | Lion Seeds Ltd. |
| 45 | LS2502 | Lion Seeds Ltd. |
| 46 | KWS0023 | KWS SAAT SE |
| 47 | KWS3473 | KWS SAAT SE |
| 48 | KWS4502 | KWS SAAT SE |
